# Binding Molecules in Tick Saliva for Targeting Host Cytokines, Chemokines, and Beyond

**DOI:** 10.3390/biom14121647

**Published:** 2024-12-21

**Authors:** Chamberttan Souza Desidério, Victor Hugo Palhares Flávio-Reis, Yago Marcos Pessoa-Gonçalves, Rafael Destro Rosa Tiveron, Helioswilton Sales-Campos, Andrei Giacchetto Felice, Siomar de Castro Soares, Rhainer Guillermo-Ferreira, Wellington Francisco Rodrigues, Carlo José Freire Oliveira

**Affiliations:** 1 Department of Microbiology, Immunology and Parasitology, Institute of Biological and Natural Sciences, Federal University of Triângulo Mineiro, Uberaba 38025-180, MG, Brazil; chamberttan_sd@hotmail.com (C.S.D.); victorvg10@msn.com (V.H.P.F.-R.); yagompg98@gmail.com (Y.M.P.-G.); rafael.tiveron@uftm.edu.br (R.D.R.T.); andreigf@hotmail.com (A.G.F.); siomar.soares@uftm.edu.br (S.d.C.S.); wellington.frodrigues@hotmail.com (W.F.R.); 2 Department of Bioscience and Technology, Institute of Tropical Pathology and Public Health, Federal University of Goiás, Goiania 74605-050, GO, Brazil; tonsales@ufg.br; 3 LESTES Laboratory, Department of Biological Sciences, Federal University of Triângulo Mineiro, Uberaba 38025-180, MG, Brazil; rhainer.ferreira@uftm.edu.br

**Keywords:** ticks, saliva, binding molecules, immuno-mediated disorders

## Abstract

Ticks have coevolved with their hosts over millions of years, developing the ability to evade hemostatic, inflammatory, and immunological responses. Salivary molecules from these vectors bind to cytokines, chemokines, antibodies, complement system proteins, vasodilators, and molecules involved in coagulation and platelet aggregation, among others, inhibiting or blocking their activities. Initially studied to understand the complexities of tick–host interactions, these molecules have been more recently recognized for their potential clinical applications. Their ability to bind to soluble molecules and modulate important physiological systems, such as immunity, hemostasis, and coagulation, positions them as promising candidates for future therapeutic development. This review aims to identify the binding molecules present in tick saliva, determine their primary targets, and explore the tick species involved in these processes. By associating the binding molecules, the molecules to which they bind, and the effect caused, the review provides a basis for understanding how these molecules can contribute to possible future advances in clinical applications.

## 1. Introduction

The saliva of blood-feeding arthropods, such as ticks, plays an essential role in hematophagy, since it has a cocktail of biomolecules with tactical anti-hemostatic, anti-inflammatory, and immunosuppressive properties. These salivary activities include the suppression of innate and adaptive immune responses and modulation of detection and response mechanisms during attachment and feeding, even though tissue damage itself can act as a trigger for immune responses. Evidence suggests that the molecules not only facilitate ectoparasitism but also the initial transmission and the course of the infection, promoting the persistence and pathogenicity of the arthropod-borne pathogens. All these effects are possible due to the quantity and diversity of bioactive molecules present in saliva that exert important effects immediately after attachment and remain until the tick detaches itself from the host’s skin. These bioactive molecules include proteins, peptides, carbohydrates, nucleosides, and lipids [1].

In fact [1], the role of tick saliva in immunomodulation is well-documented. Several studies, especially those carried out in the last two decades, have shown that salivary molecules play a role in modulating the biology of cells such as neutrophils, macrophages, dendritic cells, mast cells, basophils, B lymphocytes, and T lymphocytes. In addition to cell targets, these molecules have the ability to modulate several soluble host molecules, including cytokines, chemokines, proteins of the complement system, histamine, and antibodies, which is notable. For example, recent research indicates that certain salivary proteins, such as evasins and ixostatins, can inhibit chemokines and matrix metalloproteinases, respectively, suggesting potential applications in the treatment of inflammatory and autoimmune diseases [1,2].

This arsenal of bioactive molecules capable of modulating the host’s immune system has garnered significant interest from the scientific community due to the potential for developing new immunomodulatory therapies. At this point, those molecules capable of binding to key molecules of the immune and hemostatic systems gain prominence given their potential effect, low immunogenicity, and ease of application after isolation and identification. In other words, many research projects indicate that the molecules present in tick saliva could be exploited as prototypes for the creation of innovative therapies that not only treat immune system-related diseases but may also be used in the prevention of tick-borne infections. These biomolecules may open new avenues in the field of immunotherapy, offering more targeted and less toxic therapeutic approaches compared to conventional treatments [1,3,4]. Well-characterized classes of these ligands include ligands of chemokines (e.g., evasins), proteins of the complement system, antibodies, and proteins of the coagulation system. There are also less-studied classes of molecules with enormous clinical potential in immune response modulation, such as cytokine-binding [2,5,6,7].

This narrative review aims to summarize the existing literature on the binding molecules found in tick saliva. The primary objective of this study was to identify and compile studies that report binding molecules present in tick saliva while exploring the tick species involved in these processes. Furthermore, we sought to summarize the study designs and scientific findings related to binding molecules identified in tick saliva. Additionally, we aimed to identify gaps in the current literature and provide recommendations for future investigations into the potential applications of these molecules, with the goal of advancing scientific knowledge in this field.

## 2. Materials and Methods

### 2.1. Study Design

• A narrative review was conducted following the Preferred Reporting Items for Systematic Reviews and Meta-Analyses for Scoping Reviews (PRISMA-ScR—https://www.prisma-statement.org/ accessed on 30 August 2024) guidelines. The narrative review was registered on the Open Science Framework (OSF), and the registration can be accessed at the following link: https://osf.io/pn2t3 (accessed on 7 October 2024). The research question was developed using the PICo framework: “What is the current state of the art in the literature on binding molecules found in tick saliva, including their specific targets and associated biological actions?”
• Population (P): Ticks (any species).
• Interest (I): Binding molecules in tick saliva, their key targets, the tick species involved, and the spatial and temporal distribution of studies.
• Context (Co): Feeding context (saliva).

### 2.2. Search Strategy and Data Sources

To conduct this narrative review, comprehensive searches were conducted on 23 August 2024 to identify relevant studies. Searches were performed across electronic databases, including the National Library of Medicine of the United States of America National Institutes of Health (PubMed^®^), Web of Science™, and Excerpta Medica dataBASE (EMBASE). Additionally, studies were identified through a thorough examination of the references cited in the selected articles to ensure no essential information was overlooked. The search terms are outlined in Box S1 (Supplementary Material). No language restrictions were applied. The retrieved studies were imported into Zotero version 6.0.36 (Corporation for Digital Scholarship, Vienna, VA, USA), and duplicate records were removed to ensure data integrity. After removing duplicates, the records were imported into Rayyan.ai (Qatar Foundation, Cambridge, MA, USA), and the titles and abstracts were screened blinded. Subsequently, the articles were fully read and assessed for eligibility, and data were extracted for qualitative analyses of the included articles. These analyses were conducted by two reviewers (CSD and VHPFR) and disagreements were resolved by a third author (YMPG).

### 2.3. Inclusion and Exclusion Criteria

The inclusion criteria were as follows: (1) ticks of any species, (2) molecule with binding capacity, (3) present in the tick’s saliva. Exclusion criteria were as follows: (1) animals other than ticks, (2) molecules with no binding capacity, or present in tissues other than saliva, and (3) abstracts, reviews, or conference proceedings.

### 2.4. Extraction of Data

The data from all included studies were extracted by CSD, YMPG, and VHPFR with a structured data extraction sheet using Microsoft Excel 2016 version 2403 (Microsoft Corporation, Albuquerque, NM, USA).

The extracted data comprehensively covered six main characteristics of each study. These included the tick species, the binding molecule, the target of that molecule, the family of the ligand, the mechanism of action, and the final biological effect of this binding.

## 3. Results

### Tick Species and Molecules with Binding Properties

**Figure 1:**
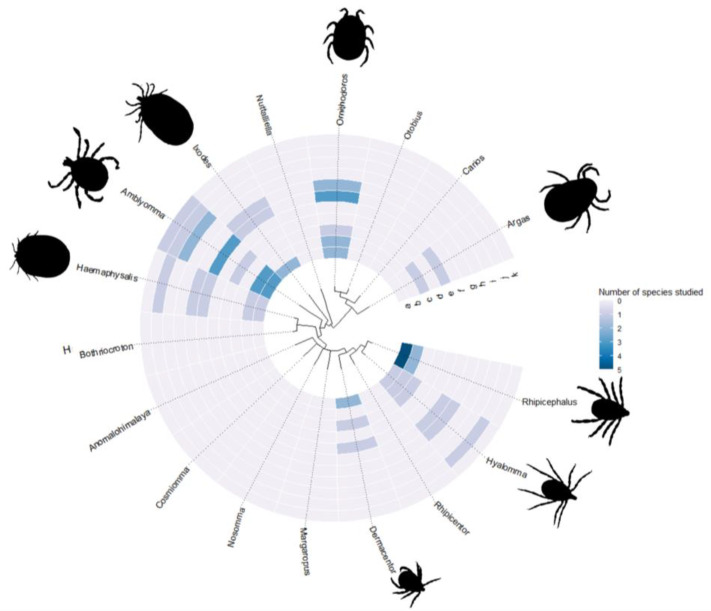
Tick families represented Tick families represented and their respective clades; the shades of blue represent the number of studies per molecule per clade, the letters represent the molecules presented in Table 1.

Regarding the tick species identified in the review, Table 1 shows the presence of twenty-two different species, eighteen of which are classified as hard ticks and four as soft ticks. Figure 1 visualizes the tick families and the number of species studied within each family, as presented in the study. Together, these species produce more than 60 binding molecules, some of which have not yet been assigned specific nomenclature. There is a predominance of the class of evasins, which primarily bind to chemokines; this class is extensively studied, as our findings highlight, with 54% of the studies demonstrating molecules that target chemokines. Other systems are also regulated; the coagulation system is primarily regulated through thrombin (around 30% of the species show molecules that bind to thrombin), and the complement system (eight molecules were identified as binding to complement system elements). It is also worth noting the presence of cytokine-binding molecules (36% of the species show studies involving at least one cytokine), a class that has not been extensively studied, though the available research highlights the potential regulation of these cytokines by saliva or salivary gland extracts.

Sting in several aspects, whether in understanding the host–parasite relationship or in potential applications. However, the number of molecules that are not yet known and isolated may provide data with countless other possibilities. Studies of the genome, transcriptome, lipidome, and proteome of the saliva of ticks and other hematophagous arthropods indicate that the number of bioactive molecules may be in the thousands and many more studies still need to be carried out in the near future [103,104].

The more than 60 identified binding molecules include various classes, such as chemokine inhibitors (evasins), molecules that inhibit components of the complement system, and coagulation factor inhibitors. Evasins have been extensively studied, particularly for their ability to bind and neutralize chemokines, which play a critical role in recruiting leukocytes to sites of infection or injury [54,55,64,105,106,107]. Among the molecules found in tick saliva, evasins are among the most frequently identified. These molecules specifically bind to host chemokines, neutralizing their activity and, consequently, the signaling of chemokine receptors [5,6]. This neutralization results in several significant consequences for the host, including reduced leukocyte migration to sites of tissue injury, potentially diminishing the inflammatory response and partially evading host immune defenses, thereby facilitating prolonged feeding. Additionally, it impairs immune responses, increases susceptibility to infections, disrupts processes such as angiogenesis and wound healing, and creates an environment conducive to pathogen transmission, aiding in the establishment of bacteria and viruses within the host [2]. Despite the extensive research on evasins, they belong to a large and diverse class, including many lesser-known members. Additionally, their detailed mechanisms of action, coevolution with host chemokines, and the immune responses they might trigger in humans remain incompletely understood. A more comprehensive understanding of evasins and their mechanisms could pave the way for the development of more effective anti-inflammatory therapies, potentially without the side effects associated with currently available treatments.

In addition to the most frequently reported molecule, various other molecules have also been identified. Among the molecules and their mechanisms, the following are noteworthy: suppression of immune system factors, thrombin inhibitors, histamine inhibitors, insulin sequestrants, serotonin inhibitors, coagulation factor inhibitors, platelet aggregation inhibitors, papain inhibitors, trypsin inhibitors, kallikrein inhibitors, and plasmin inhibitors. Molecules responsible for the suppression of immune system factors, such as B-cell inhibitory factor (BIF) [40] and I. ricinus anti-complement (IRAC I and II) [44], primarily target the host’s complement system or directly affect lymphocytic immune responses. Additionally, the diversity of other inhibitory molecules represents mechanisms of action that remain partially understood but demonstrate clear inhibitory activity. For example, Monotonin binds to host serotonin, effectively inhibiting it [26]. This vast array of molecules found in tick saliva highlights the immunogenic and immunomodulatory potential of this parasite.

Tick saliva contains a variety of immunomodulatory proteins and peptides that inhibit or redirect the host’s immune response, facilitating prolonged feeding and pathogen transmission [108]. Discoveries about the immunosuppressive properties of salivary molecules, which can inhibit the activation of cytokines such as IL-17 and IFN-γ, have broadened the scope of investigations to understand how these interactions can be therapeutically exploited or prevented by vaccines [109].

Studies involving tick salivary gland extracts are more abundant, likely because saliva extraction and characterization methods are relatively accessible, allowing for detailed analyses of its content. Assays such as ELISA and Western blot analysis have been fundamental in investigating the ability of these molecules to bind to cytokine receptors and thus modulate immune responses. However, the functional characterization of salivary molecules is still in its early stages, especially when compared to other fields of parasitic immunology. This indicates that, despite growing interest, the topic remains poorly understood and there are vast knowledge gaps. Nonetheless, the recent increase in publications, particularly after 2008, offers an optimistic outlook for future discoveries. A key avenue for expanding this field is the development of new molecular tools for the functional characterization of salivary proteins and their interactions with host cells. With the advent of next-generation sequencing and proteomics technologies, future studies may provide a broader and more detailed view of the functional diversity of compounds found in the salivary gland extract of different tick species [103,104,110].

Several studies have demonstrated that molecules produced in the saliva of hard ticks significantly modulate the host immune response. These molecules inhibit cytokine production and T cell activation, thereby compromising the host’s ability to mount an effective inflammatory response. Importantly, these molecules are required to induce prolonged immunosuppression to sustain the extended feeding periods typical of hard ticks. In contrast, soft ticks, which have shorter feeding durations, also produce bioactive molecules; however, their need for complex immunomodulation is reduced. Consequently, the primary function of soft tick saliva is to inhibit blood clotting and vasoconstriction during feeding, with less emphasis on prolonged immunosuppression.

Another critical aspect is the role of molecules that bind to coagulation and complement factors. These systems are primary targets for ticks, as blood clotting and complement activation represent immediate barriers to successful feeding. Molecules that inhibit coagulation, such as salpin from ticks of the genus *Ixodes*, and complement inhibitors, such as Isac from *Ixodes scapularis*, are essential for enabling prolonged and effective feeding. The complement system, which plays a key role in host defense against pathogens, is also inhibited by these molecules, facilitating pathogen transmission. For example, the inhibition of complement activity can aid the transmission of *Borrelia burgdorferi*, the causative agent of Lyme disease. In summary, this review highlights the diverse array of immunomodulatory molecules produced by ticks, with many already well-characterized and others yet to be explored. Studying these molecules not only enhances our understanding of tick biology but also paves the way for the development of novel therapeutic approaches targeting the modulation of the immune system.

The diversity of tick species and their immunomodulatory salivary molecules underscore the importance of understanding the distinct evolutionary strategies these ectoparasites have developed to feed and evade host immunological detection. Hard ticks, such as those of the genus *Amblyomma*, are renowned for their prolonged interactions with hosts, necessitating a diverse biochemical arsenal to inhibit immune responses and facilitate pathogen transmission. The greater number of studies on molecules from hard tick species capable of binding to proteins, receptors, and molecules involved in the host immune response is likely attributed to their extended feeding periods, which can last from days to weeks. In contrast, soft ticks feed for only a few hours, resulting in reduced host exposure and, consequently, the production of fewer immunomodulatory molecules or molecules less associated with prolonged immunosuppression [54,105]. Finally, it is worth mentioning that there are more than 800 species of ticks and if we consider that these different species suffer different environmental pressures in different parts of the world, it is to be expected that much still needs to be studied and many other molecules will still be discovered, identified, and characterized.

It is important to emphasize that the potential of cytokine-binding molecules remains underexplored, representing a class with fewer studies but great promise for therapeutic applications in disease treatment. When considering the immunomodulatory role of tick saliva, it is crucial to acknowledge specific nuances, even within the same tick family, where distinct mechanisms of action may exist. Numerous studies have demonstrated the immunomodulatory capacity of tick saliva; however, different species may modulate the immune response in varying ways. For example, studies on the saliva of *Ixodes scapularis* show the secretion of molecules that suppress T cell activation and inhibit the production of pro-inflammatory cytokines. In contrast, ticks such as *Amblyomma sculptum* secrete saliva with anti-inflammatory properties, capable of modulating cytokines such as IL-4, IL-17, and IFN-γ. Cytokines like IL-4, IL-17, and IFN-γ play central roles in regulating both innate and adaptive immune responses. The modulation of these cytokines by tick saliva represents a promising avenue, particularly for the development of immunosuppressive therapies based on natural molecules [106,107,111]. The lack of standardized nomenclature for some of these molecules suggests that many are still in the early stages of characterization, highlighting their vast potential for future research.

## 4. Conclusions

This narrative review provides a comprehensive overview of the binding molecules present in tick saliva. It is noteworthy that, although significant advances have been made in identifying and characterizing these molecules, substantial knowledge gaps remain, particularly regarding those that modulate cytokines. Ticks possess a remarkable diversity of molecules that facilitate feeding and infecting their hosts. While numerous molecules have been documented, many still lack fully understood mechanisms of action. This vast molecular diversity in tick saliva represents a promising area of research, not only to enhance our understanding of tick biology and its interactions with hosts, but also to develop new therapeutic approaches based on immune modulation and coagulation system regulation, as highlighted by several studies discussed here. Future research focusing on the functional diversity of these molecules, especially those involved in cytokine inhibition, has the potential to yield significant discoveries. These findings may contribute to innovative strategies for combating inflammatory and autoimmune diseases. The potential of these molecules for immune modulation is vast, and continued research is likely to uncover new therapeutic opportunities and applications in human health.

## Figures and Tables

**Table 1:** Overview of binding molecules present in tick saliva and their targets, actions and effects. Abbreviations and respective names are in a table in the supplementary material.

Tick	Binding Molecule	Target	Action	Effect	Reference
*Amblyomma americanum* (Hard Tick)	Calreticulin (AamCRT)	C1q	Binds to C1q	Binds to C1q, but the progression of the pathway is not affected. This contrasts with other parasite CRTs that block the pathway	[8]
	Insulin-like Growth Factor Binding Protein-related Protein 1 (Aam IGFBP-rP1)	Insulin	Binds to insulin	Insulin is sequestered at the feeding site by the tick’s native protein, which consequently interferes with the host’s tissue repair mechanisms	[9]
	Macrophage migration inhibitory factor (HLMIF)	Immunoregulatory cytokine	–	Inhibits macrophage migration	[10]
	P1243	CC chemokine-binding proteins	Binds to CC chemokines	Neutralizes chemokine activity	[11]
	Serpin 19 (AAS19)	Heparan sulfate/heparin (HS)	Binds to HS	Promotes inhibits against Thrombin, IXa, Xa, XIIa, trypsin, kallikrein, papain, and plasmin	[12,13,14]
*Amblyomma cajennense* (Hard Tick)	Amblyomin-X	Factor Xa; Ubiquitin-proteasome system (UBS)	Binds to factor Xa and UBS	Inhibits factor Xa, prothrombinase, and tenase, promoting anticoagulation. Exhibits anti-tumoral activity by promoting apoptosis and reducing tumor cells in vivo	[15,16,17]
	EVA-ACA1001 (Class A3 evasin)	CC chemokines	Binds to 21 of 24 human CC chemokines	Inhibits the activation of chemokine receptors, promoting suppression of leukocyte recruitment and inflammation	[18]
	Sculptin	Thrombin	Binds to thrombin	Presents antithrombotic properties	[19,20]
*Amblyomma sculptum* (Hard Tick)	As8.9kDa	–	–	Inhibits classical complement pathway, factor Xa, thrombin, and trypsin	[21]
	AsBasicTail	–	–	Inhibits factor Xa, thrombin, trypsin, and alternative complement pathway	[21]
	AsKunitz	–	–	Inhibits thrombin and classical complement pathway	[21]
*Amblyomma variegatum* (Hard Tick)	–	CXCL-8, CCL2, CCL3, CCL5, CCL11, IL-2, and IL-4	Binds to CXCL-8, CCL2, CCL3, CCL5, CCL11, IL-2, and IL-4	Inhibits cytokines and chemokines	[22]
	–	IL-8, MCP-1, MIP-1alpha, RANTES, eotaxin, IL-2, and IL-4.	Binds to IL-8, MCP-1, MIP-1alpha, RANTES, eotaxin, IL-2, and IL-4	Inhibits cytokines and chemokines	[7]
*Argas monolakensis* (Soft Tick)	Monomine	Histamine	Binds to histamine	Inhibits histamine	[8]
	Monotonin	5-hydroxytryptamine (serotonin)	Binds to serotonin	Inhibits serotonin	[8]
	Monogrin	Fibrinogen receptor GPIIbIIIa	Binds to the GPIIbIIIa molecule	Inhibition of platelet aggregation	[23]
	Monobin	Thrombin	Binds to thrombin	Inhibits thrombin	[23]
	AM-33	LTC4 e LTD4	bind cysteinyl leukotrienes	Modulation of the inflammatory response at the tick feeding site	[24]
*Dermacentor marginatus* (Hard Tick)	Hemelipoglycoprotein	Fibrinogen-related proteins	Binds to fibrinogen-related proteins	Involvements in tick innate immunity	[25,26]
*Ornithodoros savignyi* (Soft Tick)	TAP	Factor Xa (fXa)	Binds to activated factor Xa (fXa)	Inhibits factor Xa	[27]
*Dermacentor variabilis* (Hard Tick)	Variabilin	Glycoprotein IIb-IIIa (GPIIb-IIIa) complex	Binds to the glycoprotein through the arginine-glycine-aspartic acid sequence present in Variabilin	Inhibition of platelet aggregation	[28]
*Ornithodoros moubata* (Soft Tick)	OMCI	C5	OmCI directly interacts with C5	Inhibition of the complement activation pathway	[29]
*Ixodes scapularis* (Hard Tick)	Sialostatin L	Cathepsins	Formation of a stable complex between Cystatin and the cathepsins	Suppression of inflammation and the host’s immune response	[30]
*Dermacentor reticulatus* (Hard Tick)	Anti-IL-8	IL-8	Binds to IL-8	Inhibits neutrophil chemoattraction and activation	[31,32]
	SHBP	Histamine and serotonin	Binds to histamine and serotonin	Inhibits the function of the targets	[33]
	–	CXCL-8, CCL2, CCL3, CCL5, CCL11, IL-2, IL-4	Binds to CXCL-8, CCL2, CCL3, CCL5, CCL11, IL-2, and IL-4	Inhibits cytokines and chemokines	[22]
	–	IL-8, MCP-1, MIP-1alpha, RANTES, eotaxin, IL-2 and IL-4.	Binds to IL-8, MCP-1, MIP-1alpha, RANTES, eotaxin, IL-2, and IL-4	Inhibits cytokines and chemokines	[7]
*Haemaphysalis longicornis* (Hard Tick)	Haemaphysalin	Factor XII/XIIa; high molecular weight kininogen (HK)	Binds to HK; inhibits the kallikrein-kinin system	Interferes with the association of factor XII and the prekallikrein-HK complex; inhibits the intrinsic blood coagulation pathway	[34,35]
	HIDfsin2	Lipopolysaccharide (LPS)	Binds to LPS, promoting depolymerization of LPS micelles into smaller particles	Enhances the activation of the nuclear factor NF-κB and IFN-I signaling pathways, which are downstream of TLR4	[36,37]
	Macrophage migration inhibitory factor (HLMIF)	Immunoregulatory cytokine	–	Inhibits macrophage migration	[38]
	Longistatin	EF-hand Ca^++^	Binds to EF-hand Ca^++^	The expression of the gene for Longistatin production increases primarily during feeding, indicating an important role in the blood-meal	[39,40]
		Fibrinogen	Binds to fibrinogen, promoting hydrolysis of the α, β, and γ chains; activates plasminogen	Delays fibrin clot formation; binds to fibrin meshwork; activates fibrin clot-bound plasminogen (plasmin); induces lysis of fibrin clots and platelet-rich thrombi	[40,41]
		Receptor for Advanced Glycation end Products (RAGE)	Binds to RAGE V domain; RAGE antagonist	Attenuates cellular oxidative stress and prevents NF-κB translocation; reduces adhesion molecule and cytokine production; suppresses tick-bite associated inflammation	[42]
	Madanin 1 and 2	Thrombin	Binds to thrombin	Inhibits conversion of fibrinogen into fibrin by thrombin, thrombin-catalyzed activation of factor V and factor VIII, and thrombin-induced aggregation of platelets	[43]
*Hyalomma asiaticum* (Hard Tick)	Ha24	Histamine	Binds to histamine	Inhibits the action of histamine	[44]
	B-cell inhibitory factor (BIF)	Lymphocyte B	Inhibits lipopolysaccharide-induced B-cell proliferation	Inhibits lymphocyte B	[45]
*Hyalomma dromedarii* (Hard Tick)	rDromaserpin	Thrombin	Binds to thrombin	Inhibits thrombin, kallikrein, factor XIa, factor XIIa, and thrombin-induced platelet aggregation	[46]
	Camel tick salivary gland thrombin inhibitor	Thrombin	Binds to thrombin	Inhibits thrombin	[47]
*Ixodes persulcatus* (Hard Tick)	I persulcatus cystatin (JpIpcy)	Cathepsin L	Binds to cathepsin L	–	[48]
*Ixodes Ricinus* (Hard Tick)	I. ricinus anticomplement (IRAC I and II)	–	Inhibits C3b binding; accelerates uncoupling of factor Bb from C3 convertase	Inhibits alternative complement pathway	[49]
	I ricinus serpin-2 (IRS-2)	Cathepesin G; Chymase; Thrombin	Inhibits cathepesin G, chymase and thrombin	Inhibits cathepesin G- and thrombin-induced platelet aggregation; inhibits edema formation and influx of neutrophils in the inflamed tissue	[50]
	IxACs	Properdin	Binds to properdin	Inhibits C3 convertase and the alternative complement pathway	[51]
	Ixodes ricinus leukotriene B4-binding protein (Ir-LBP)	Leukotriene B4	Binds to leukotriene B4	Inhibits neutrophil chemotaxis and delays LTB4-induced apoptosis	[52,53]
	Iripin-3	–	Inhibits kallikrein and matriptase	Decrease in CD4^+^ T lymphocyte proliferation, suppression of Th1 response, and inhibition of IL-6 in macrophages	[54]
	Iripin-8	–	Inhibits plasmin, trypsin, and kallikrein	Inhibits multiple proteases involved in blood coagulation and blocks the intrinsic and common pathways of coagulation	[55]
	Ixodes ricinus immunosuppressor protein (IRIS)	–	–	Inhibits IFN-γ production by T lymphocytes and inhibits IL-6 and TNF-α production by macrophages	[56]
	P1156	CXC chemokine-binding proteins	Binds to CXC chemokines	Neutralizes chemokine activity	[11]
	Salp15 Iric-1	CD4 coreceptor	Repression of calcium fluxes triggered by TCR ligation resulting in lower production of IL-2	Inhibits CD4+ T cell activation	[57]
	TNF-α-inhibitory	TNF- α	Binds to TNF- α	Inhibits the cytokine effect	[58]
	–	CXCL-8, CCL2, CCL3, CCL5, CCL11, IL-2, IL-4	Binds to CXCL-8, CCL2, CCL3, CCL5, CCL11, IL-2, and IL-4	Inhibits cytokines and chemokines	[22]
	–	IL-8, MCP-1, MIP-1alpha, RANTES, eotaxin, IL-2 and IL-4.	Binds to IL-8, MCP-1, MIP-1alpha, RANTES, eotaxin, IL-2, and IL-4	Inhibits cytokines and chemokines	[7]
*Ixodes scapularis* (Hard Tick)	IL-2 Binding Protein (IL-2BP)	IL-2	Binds to IL-2	Suppresses T cell proliferation and activity of others immune effector cells that are responsive to IL-2 stimulation	[59]
	I. scapulari salivary anticomplement (Isac)	–	Inhibits C3b binding; accelerates uncoupling of factor Bb from C3 convertase	Inhibits alternative complement pathway	[60]
	I. scapularis serpin 17 (IxsS 17)	–	–	Inhibits host innate immune system proteases and blood clotting (factor Xa and Xia); interacts with complement system	[61]
	Ixolaris	–	Tissue factor pathway inhibitor	Inhibits the activation of factor X triggered by factor VIIa/tissue factor.	[62]
	Ixonnexin	–	Supports the interaction of plasminogen with t-PA	Promotes fibrinolysis	[63]
	Salp9Pac	–	Interacts with mannan binding lectin-associated serine proteases and factor Xa	Inhibits lectin complement pathway and coagulation cascade	[64,65]
	Salp14	Man-nose-binding lectin	Interacts with mannose binding lectin–associated serine proteases and factor Xa	Inhibits lectin complement pathway and coagulation cascade	[64,65]
	Salp15	CD4 coreceptor	Repression of calcium fluxes triggered by TCR ligation resulting in lower production of IL-2	Inhibits CD4^+^ T cell activation	[66,67]
	Salp20	Properdin	Binds and displaces properdin from C3 convertase	Inhibits alternative complement pathway	[68,69]
	Sialostatin L2	Annexin A2	Binds to annexin A2	Inhibits NLRC4-Mediated inflammasome activation	[70,71]
	Tick histamine release factor (tHRF)	Basophils	Binds to basophils	Stimulates histamine release; modulates vascular permeability and increase blood flow to the tick bite-site	[72]
	Tick Salivary Lectin Pathway Inhibitor (TSLPI)	Mannose-binding lectin	Inhibits mannose-binding lectin	Inhibits lectin complement pathway	[73]
*Ornithodoros moubata* (Soft Tick)	Disagregin	αIIbβ3 integrin	Binds to αIIbβ3 integrin	Inhibits platelet aggregation induced by AD, collagen, epinephrine, platelet-activating factor, thrombin, and the thrombin receptor peptide SFLLRNPNDKYEPF	[74,75]
	Enolase	Plasminogen	Binds to plasminogen	Transforms plasminogen into plasmin, promoting fibrinolysis and degradation of extracellular matrix material	[76]
	Moubatin	–	Scavenges of thromboxane A2	Inhibits collagen-induced platelet aggregation	[77,78]
	OmCI	C5	Binds to C5	Inhibits complement system	[79]
	Ornithodorin	Thrombin	Binds to thrombin	Inhibits thrombin-induced platelet aggregation	[80]
*Ornithodoros savignyi* (Soft Tick)	BSAP1 and BSAP2	–	–	Inhibits extrinsic blood coagulation pathway	[81]
	Savignin	Thrombin	Binds to thrombin	Inhibits thrombin-induced platelet aggregation	[82,83]
	Savignygrin	αIIbβ3 integrin	Binds to αIIbβ3 integrin	Inhibits platelet aggregation induced by ADP, collagen, the thrombin receptor-activating peptide, and epinephrine	[84]
	TSGP2 and TSGP3	Leukotriene B4 and C5	Binds to leukotriene B4 and C5	Inhibits complement system and neutrophil chemotaxis	[78,85]
*Ornithodoros turicata* (Soft Tick)	Ornithodoros turicata lipocalin-like molecule (otlip)	Histamine	Binds to histamine	Inhibits the action of histamine	[86]
*Rhipicephalus appendiculatus* (Hard Tick)	Immunoglobulin-bind proteins (IGBPs)	Immunoglobulins	Binds to immunoglobulins	Inhibits immunoglobulins	[87]
	High-affinity histamine-binding proteins (Ra-HBPs)	Histamine	Binds to histamine	Inhibits the action of histamine	[88,89]
	RaCI	C5	Binds to C5	Inhibits complement system	[90]
	–	CXCL-8, CCL2, CCL3, CCL5, CCL11, IL-2, IL-4	Binds to CXCL-8, CCL2, CCL3, CCL5, CCL11, IL-2, and IL-4	Inhibits cytokines and chemokines	[22]
*Rhipicephalus pulchellus* (Hard Tick)	CirpA1	Properdin	Binds to properdin	Inhibits the alternative complement pathway	[91]
	CirpT1	C5	Binds to C5	Inhibits complement system	[92]
	P672	CCL8	Binds to CCL chemokines	Neutralizes chemokine activity	[93]
	CirpA	Properdin	Binds to properdin	Inhibits alternative complement pathway	[91]
*Rhipicephalus haemaphysaloides* (Hard Tick)	Immunoglobulin-bind proteins (IGBPs)	Immunoglobulins	Binds to immunoglobulins	Inhibits immunoglobulins	[94]
	RH36	–	–	Suppress splenocyte proliferation and inhibits expression of IL-2, IL-12, and TNF-α	[95]
*Rhipicephalus (Boophilus) microplus* (Hard Tick)	Boophilin	Thrombin	Binds to thrombin	Inhibits thrombin	[96]
	Immunoglobulin-bind proteins (IGBPs)	Immunoglobulins	Binds to immunoglobulins	Inhibits immunoglobulins	[97]
	RmS-3, RmS-6, and RmS-17	–	–	Inhibits host immune responses by modulating mast cells and lymphocytes	[98,99]
*Rhipicephalus sanguineus* (Hard Tick)	Evasin-1	CCL3; CCL4; CCL18	Binds to CCL chemokines	Neutralizes chemokine activity	[100,101]
	Evasin-3	CXCL8; CXCL1	Binds to CXCL chemokines	Neutralizes chemokine activity	[5,6]
	Evasin-4	CCL5; CCL11	Binds to CCL chemokines	Neutralizes chemokine activity	[5,102]

## Data Availability

Not applicable.

## Supplementary Materials

The following supporting information can be downloaded at: https://www.mdpi.com/article/10.3390/biom14121647/s1, Box S1. Keywords and search terms were used to identify studies involving binding molecules in tick saliva. Figure S1. PRISMA flow diagram describing the study selection process. Figure S2. Distribution of selected studies by country and/or region, the number inside the balls represents the number of articles from that country/region in the study. Figure S3. Distribution of the number of published works found in the review over the years. Table S1. List of abbreviations used within the text, alongside their corresponding definitions.
