# Supplementary material for: Binding Molecules in Tick Saliva for Targeting Host Cytokines, Chemokines, and Beyond
Source: Biomolecules. 2024 Dec 21;14(12):1647. doi: 10.3390/biom14121647 (PMC11674731; doi:10.3390/biom14121647)
Supplement: Supplementary file 1 [file biomolecules-14-01647-s001.zip › biomolecules-3270693-supplementary.pdf]

biomolecules

MDPI

Review

Binding Molecules in Ticks for Targeting Host Cytokines, Chemokines and Beyond

Chamberttan Souza Desidério [1,†], Victor Hugo Palhares Flávio-Reis [1,†], Yago Marcos Pessoa-Gonçalves [1], Rafael Destro Rosa Tiveron [1], Helioswilton Sales-Campos [2], Andrei Giacchetto Felice [1], Siomar de Castro Soares [1], Rhainer Guillermo-Ferreira [3], Wellington Francisco Rodrigues [1] and Carlo José Freire Oliveira [1,*]

[1] Department of Microbiology, Immunology and Parasitology, Institute of Biological and Natural Sciences, Federal University of Triângulo Mineiro, Uberaba, Minas Gerais, Brazil

[2] Department of Bioscience and Technology, Institute of Tropical Pathology and Public Health, Federal University of Goiás, Goiania, Goiás, Brazil

[3] LESTES Laboratory, Department of Biological Sciences, Federal University of Triângulo Mineiro, Uberaba, Minas Gerais, Brazil

† These authors contributed equally to this work.

* Correspondence: carlo.oliveira@uftm.edu.br; Tel.: +55-(034) -98856-7251

Supplementary Materials

Box S1. Keywords and search terms were used to identify studies involving binding molecules in tick saliva.

• For ticks the following keywords are used: "Ticks" OR "Tick" OR "Ixodida" OR "Tick Bite" OR "Tick Bites" OR "Argasidae"
• To delineate the saliva from ticks the following keywords are utilized: "Saliva" OR "Salivas" OR "Salivary" OR "Salivary Proteins and Peptides"
• To specify the binding molecules the following keywords are utilized: "Ligands" OR "Ligand" OR "Bind" OR "Target"
• Finally, these searches were combined to determine the final search: ("Ticks" OR "Tick" OR "Ixodida" OR "Tick Bite" OR "Tick Bites" OR "Argasidae") AND ("Saliva" OR "Salivas" OR "Salivary" OR "Salivary Proteins and Peptides") AND ("Ligands" OR "Ligand" OR "Bind" OR "Target")

The bibliographic search resulted in 787 manuscripts in three different databases, and after removing duplicates and those that did not meet the eligibility criteria (n = 358), 114 were considered for full-text review. After reading the full text, 4 articles were excluded as they involved animals other than ticks, 6 articles lacked a clear study design, and 8 articles did not address tick saliva components. This left 96 articles included in the review. Figure 1 presents a PRISMA flow diagram outlining the study selection process.

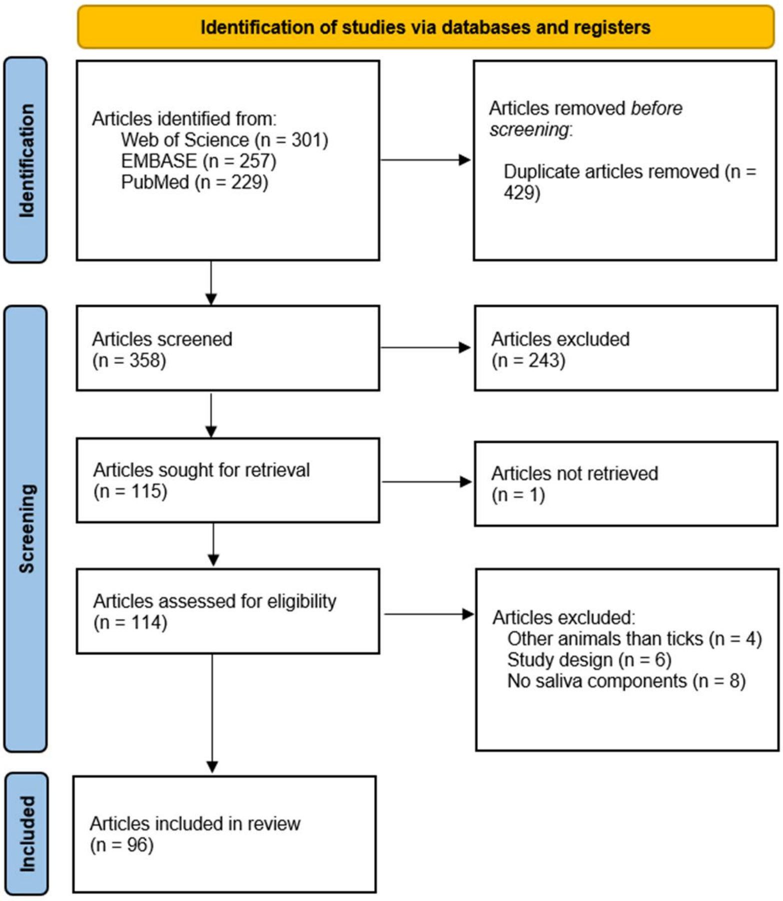

Figure S1. PRISMA flow diagram describing the study selection process.

Table S1 presents the geographic distribution of the selected studies, along with the type of study and the methodology used. Regarding geographic distribution, 3 studies involve collaboration between two countries (Brazil and Japan, the United States and the Netherlands, and the United States and South Africa). The articles conducted within a single country show a predominance of research in the U.S. (n=25), totaling 27 when including the collaborative studies. As shown in Figure 2, the United States has more than twice the number of studies compared to the countries in second place. Brazil follows with 10 individual studies, and the United Kingdom with 9. When analyzing the distribution of studies across continents, Europe includes 8 different countries researching tick saliva-binding molecules, while the Americas have only two countries (Brazil and the United States) conducting this type of research; however, these countries are involved in nearly 40% of the studies (n=38). The selected studies employ a variety of approaches— in vitro, in vivo, and in silico—covering a wide range of saliva analysis. These studies utilize various methodologies, with a predominance of molecular biology techniques (PCR, RT-PCR, and Western Blotting).

Table S1. The following table provides a comprehensive list of abbreviations used within the text, alongside their corresponding definitions.

Abbreviation	Meaning
IL - 8	Interleukin-8
HLMIF	Macrophage migration inhibitory factor
AAS19	Serpin 19
HS	Heparan sulfate/heparin

UBS	Ubiquitin-proteasome system
CXCL-8	C-X-C Motif Chemokine Ligand 8
CCL2	C-C Motif Chemokine Ligand 2
CCL3	C-C Motif Chemokine Ligand 3
CCL5	C-C Motif Chemokine Ligand 5
CCL11	C-C Motif Chemokine Ligand 11
IL-2	Interleukin-2
IL-4	Interleukin-4
MCP-1	Monocyte Chemoattractant Protein-1
MIP-1alpha	Macrophage Inflammatory Protein-1α
RANTES	Regulated on Activation, Normal T-cell Expressed and Secreted
Eotaxin	C-C Motif Chemokine Ligand 11 (CCL11)
LPS	Lipopolysaccharide
CRT	Calreticulin
IGFBP-rP1	Insulin-like Growth Factor Binding Protein-related Protein 1
RAGE	Receptor for Advanced Glycation end Products
BIF	B-cell inhibitory fac-tor
tHRF	Tick histamine release factor
TSLPI	Tick Salivary Lectin Pathway Inhibitor
IGBPs	Immunoglobulin-bind proteins
HBPs	High-affinity histamine binding proteins

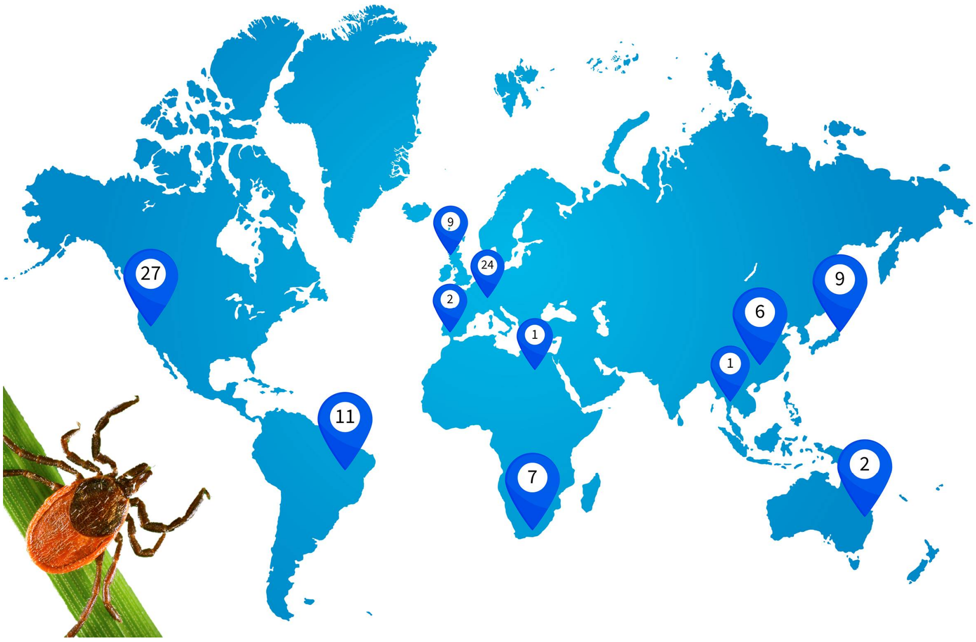

Figure S2. Distribution of selected studies by country and/or region, the number inside the balls represents the number of articles from that country/region in the study.

The studies span from 1993 to 2024, though the distribution is not homogeneous (K2 = 16.30; p = 0.0003). There were years within this time frame that had no publications on the subject. The peak of publications occurred in 2008, with 11 studies published on the topic, representing 11.3% of the total studies (Figure S2). Although this is an area of growing interest, it remains largely underexplored. Studies involving tick salivary gland extracts are more abundant than those demonstrating the effect of a specific isolated molecule that binds to components controlling the host's biology.

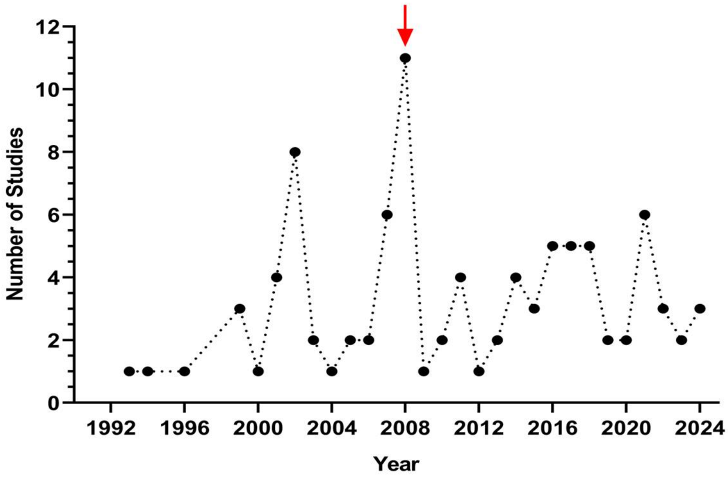

Figure S3. Distribution of the number of published works found in the review over the years.
